# Energy-based indicators and optimization of coal pillar width in gob-side entry driving

**DOI:** 10.1038/s41598-026-50873-9

**Published:** 2026-05-02

**Authors:** Yi Zhang, Qingli Gao, Wei Zhang, Jin Chen, Hengkai Wang

**Affiliations:** Shool of Mechanics and Civil Engineering, China University of Mining & Technology (Beijing), Beijing, 100083 China

**Keywords:** Gob-side entry driving, Coal pillar width, Energy-based stability analysis, Elastic strain energy, Dissipated energy, Dissipated energy ratio, Energy science and technology, Engineering, Solid Earth sciences

## Abstract

Gob-side entry driving is widely applied in deep coal mines, where rapid unloading of surrounding rock on the gob side induces stress redistribution, and the coal pillar is consequently regarded as a key load-bearing structure. The stability of the roadway is governed by the competition between elastic elastic strain energy and dissipated energy within the coal pillar. To address the difficulty of identifying stability state transition points in coal pillar width design under deep burial and weak rock conditions, this study analyzes the surrounding rock response from an energy perspective and establishes an energy analysis framework based on the coupling of elastic elastic strain energy and dissipated energy, with the dissipated energy ratio introduced as an evaluation index. Based on FLAC3D numerical simulations, the spatial distribution and evolution of elastic strain energy, dissipated energy, and dissipated energy ratio under different coal pillar widths are investigated. The results indicate that when the coal pillar width increases from 4 to 6 m, the bearing mechanism gradually shifts from plastic dissipation-dominated behavior to an elastoplastic coordinated state dominated by elastic elastic strain energy, with the dissipated energy ratio decreasing from 1 to approximately 0.67. When the width further increases to 8 ~ 14 m, elastic strain energy rapidly accumulates in the central region of the coal pillar, resulting in the formation of a pronounced energy concentration zone. Compared with traditional indicators based on stress, displacement, and plastic zone distribution, the dissipated energy ratio is more effective in characterizing. Considering energy evolution characteristics, bearing capacity, and engineering economy, a 6 m coal pillar is considered to achieve the most favorable balance under the conditions of the studied mine. Field monitoring results further verify the engineering applicability of the proposed energy-based criterion and coal pillar width optimization scheme.

## Introduction

With the continuous increase in coal mining depth, gob-side entry driving has been widely applied in deep coal mines in China as an important technique for achieving efficient extraction, conserving resources, and improving the flexibility of roadway layout^1,2^. However, under excavation-induced disturbances, rapid unloading of the surrounding rock on the gob side occurs, leading to stress redistribution, and the coal pillar is consequently regarded as a key load-bearing structure^3^. Previous studies have shown that excavation-induced unloading can result in inconsistent stress adjustment paths in surrounding rock, leading to complex and non-uniform failure mechanisms^4^.

If the coal pillar width is insufficient, penetration of the plastic zone may occur, leading to instability; conversely, an excessively wide pillar may result in unnecessary resource loss^5–7^. Therefore, under deep and complex stress environments, the determination of a reasonable coal pillar width for gob-side entry driving is of fundamental significance for ensuring long-term roadway stability and achieving safe and efficient coal mining^8,9^.

Traditional coal pillar width design mainly relies on stress distribution, the extent of the plastic zone, or empirical criteria. For example, Huo Bingjie et al.^10^ suggested that a coal pillar width of 6 m is relatively safe under extra-thick coal seam conditions; Shan Chengfang et al.^11^ reported that a 4 m coal pillar can satisfy deformation control requirements in “double-hard” coal seams; and Li Yanhe et al.^12^ proposed a reasonable pillar width of 6 m for closely spaced coal seams at depths of approximately 1000 m. These studies provide important engineering references from stress- or strength-based perspectives. However, with the increasing occurrence of surrounding rock instability in deep mining, it has been gradually recognized that the fundamental driving force of deep roadway failure is not stress itself, but the imbalance among energy input, accumulation, and dissipation^13^.

In recent years, energy-based approaches have gradually become effective methods for revealing the deformation and failure mechanisms of surrounding rock in deep mining. It has been further demonstrated by recent numerical studies that coal pillar damage and instability are closely related to the evolution of elastic strain energy accumulation and energy dissipation with increasing width-to-height ratios^14,15^. Tang Dongxu et al.^16^ indicated that elastic elastic strain energy is an important triggering factor for structural instability in gob-side roadways. Jia et al.^17^ demonstrated through theoretical analysis and numerical simulations that wide coal pillars are prone to forming energy concentration zones, thereby increasing the risk of rock bursts. Chen Dingchao et al.^18^ proposed the full-cycle evolutionary relationship between surrounding rock energy and stress, providing a quantitative basis for identifying weakened zones in deep roadways. Liu et al.^14^ developed an energy-driven failure model for roadway surrounding rock using numerical simulations and systematically analyzed the elastic elastic strain energy, energy dissipation, and energy release characteristics under different disturbance conditions, proposing corresponding energy-based classification control strategies. In addition, indicators such as deviatoric stress, distortional energy, and dissipated energy have been shown to be closely related to plastic failure and crack propagation processes^19^. Although existing studies have emphasized the role of energy in roadway stability, a unified framework capable of quantitatively describing the variation in the proportion of elastic strain energy and energy dissipation within coal pillars with increasing width is still lacking, and energy-based indicators that can be directly applied to coal pillar width determination have not yet been established.

The Changping Coal Mine is characterized by a large burial depth and a high in-situ stress environment.The roof and floor strata are mainly composed of mudstone and sandy mudstone, and are highly susceptible to water-induced softening, resulting in a significant reduction in strength and stiffness.In addition, the coal seam and surrounding rock are characterized by relatively low mechanical strength and poor structural stability.Under such geological conditions, large deformation and instability are easily induced during gob-side entry driving, particularly under the coupled effects of mining disturbance and weak rock mass behavior.Conventional evaluation methods based on stress, displacement, or plastic zone distribution are insufficient to fully characterize the progressive failure process.Therefore, a more comprehensive framework is required to describe the deformation and instability mechanisms.In this study, the 6312 working face of Changping Coal Mine is selected as the engineering background, and the coupled evolution of elastic strain energy and dissipated energy in coal pillars is investigated from an energy perspective.The energy decomposition framework dominated by spherical stress and deviatoric stress is systematically introduced into the bearing analysis of coal pillars in gob-side entry driving. Furthermore, based on the explicit finite difference method implemented in FLAC3D, the evolution laws of elastic strain energy, dissipated energy, and dissipated energy ratio under different coal pillar widths are quantitatively investigated, and the transition of the coal pillar bearing mode from plastic-dominated to elastic-dominated behavior with increasing width is identified. Finally, field monitoring data are used to verify the reliability of the analysis, and an energy-regulation-based combined support scheme is proposed.

This study aims to reinterpret the width effect and stability mechanism of coal pillars in gob-side entry driving from an energy perspective based on numerical simulation results, with emphasis on the characteristics and spatial distribution of energy evolution rather than on establishing a rigorous theory of energy conservation and transfer. The proposed approach provides a quantifiable and generalizable technical pathway for the design and support optimization of gob-side entry driving in deep coal mines.

## Energy-redistribution mechanism of the coal pillar under along-goaf excavation disturbance

### Energy variation due to surrounding-rock unloading and lateral additional loading on the coal pillar

**Fig. 1:**
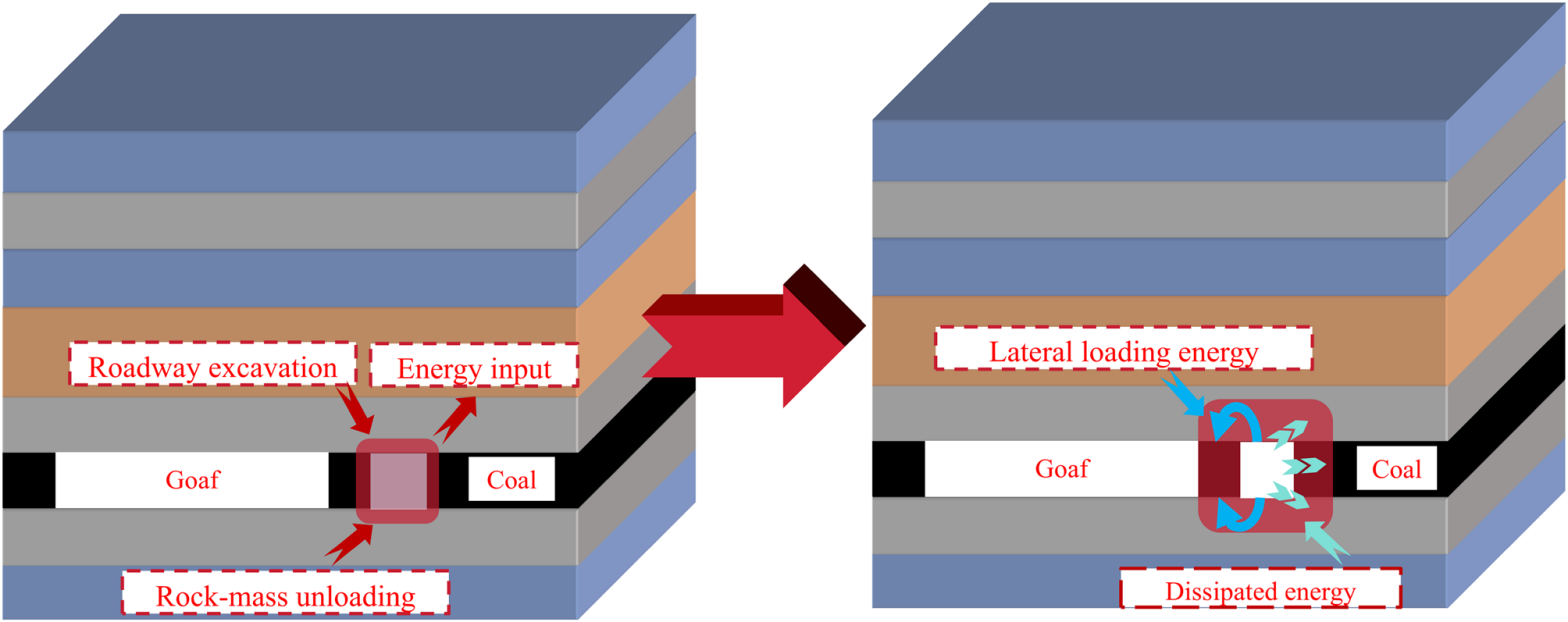
Energy evolution in gob-side roadways.

Gob-side entry driving can be regarded as a typical local unloading process. Before excavation, the coal pillar and the adjacent surrounding rock are subjected to a triaxial in-situ stress state and a certain amount of elastic strain energy is stored. When roadway excavation causes part of the surrounding rock to lose its load-bearing structure, the stress in the unloading zone is rapidly released from the initial state to nearly zero, resulting in the release of stored elastic energy and the formation of disturbance energy input. This energy is transmitted to areas adjacent to the roadway through contact interfaces and coal-rock structural interactions. This process does not involve energy input, but rather represents a redistribution of internal stored energy within the system, which can be approximately regarded as an energy source term induced by gob-side entry driving disturbances. Part of the energy released from the surrounding rock is absorbed and re-stored by the nearby coal pillar, while the remaining portion is dissipated through plastic deformation and microcrack propagation. According to the concept of energy transfer, the energy obtained from unloading can be considered as the equivalent disturbance energy *E*_*m*_ of the gob-side entry driving mechanical system, which can be reabsorbed, accumulated, or dissipated by the coal pillar and surrounding rock. In this study, the surrounding rock response process is analyzed from two aspects: the energy variation associated with local surrounding rock unloading and the energy variation of the lateral additional load acting on the coal pillar, as illustrated in Fig. 1.

Taking the unloading zone volume *V*_*u*_ as the integration domain, the energy released during a single excavation step can be expressed as:

1 $${\mathrm{E}}_{{\mathrm{m}}} = \int_{{{\mathrm{V}}_{{\mathrm{u}}} }} {\left( {U_{{{\mathrm{e}}0}} - U_{{{\mathrm{e}}1}} } \right){\mathrm{dV}}}$$

where Ue0 and Ue1 denote the elastic strain energy density of an element before and after unloading, respectively. Under quasi-static excavation conditions, kinetic energy, inertial effects, and thermal terms can be neglected.

Accordingly, the energy variation of the system can be approximately expressed as a redistribution between elastic strain energy and dissipated energy. Therefore, the system can be considered to approximately satisfy the following energy balance relationship:

2 $${\mathrm{E}}_{{\mathrm{m}}} \approx {\mathrm{E}}_{{\mathrm{c}}} + {\mathrm{E}}_{{\mathrm{r}}}$$

where *E*_*c*_ represents the energy absorbed by the coal pillar, including elastic elastic strain energy and dissipated energy within the pillar, and *E*_*r*_ denotes the energy dissipated in the intact coal and the fractured surrounding rock zones. This expression characterizes the elastic energy release induced by excavation and represents the fundamental energy source of gob-side entry driving disturbances. It is used to describe the conceptual redistribution pathway of energy between the coal pillar and surrounding rock under gob-side entry driving conditions. In the subsequent numerical simulations, quantitative analysis is mainly conducted from the perspectives of elastic strain energy and plastic dissipated energy within the coal pillar.

After unloading of the surrounding rock on the gob side, the vertical load and lateral confinement originally borne by this region are transferred to the coal pillar adjacent to the roadway, causing the major principal stress of the coal pillar to increase from *σ*_*0*_ to *σ*_*1*_.Consequently, the elastic strain energy per unit volume within the coal pillar increases, accompanied by irreversible deformations such as shear failure, strain softening, and fracture dilation, which generate plastic dissipated energy. Based on this mechanism, the energy variation of the coal pillar can be decomposed into an elastic strain energy term *U*_e_ and an energy dissipation term *W*_*d*_:

3 $${\mathrm{E}}_{{\mathrm{c}}} = U_{{\mathrm{e}}} + W_{{\mathrm{d}}}$$

The determination of a reasonable coal pillar width fundamentally depends on how externally induced disturbance energy is partitioned within the coal pillar into elastic strain energy and energy dissipation, thereby governing the formation or failure of a stable load-bearing structure consisting of an elastic core and a plastic shell. The stronger the elastic strain energy capacity, the more effectively the coal pillar can develop a stable bearing structure; conversely, a higher proportion of energy dissipation indicates a greater tendency toward damage and instability. The manner in which the disturbance energy is distributed within the coal pillar is fundamentally controlled by its stress state.

### Energy-decomposition framework and the internal evolution mechanism of the coal pillar

The internal stress state of a coal-rock mass can be decomposed into a spherical (hydrostatic) stress component and a deviatoric stress component based on the three principal stresses *σ*_*1*_, *σ*_*2*_, *σ*_*3.*_ The spherical stress does not change the volumetric shape of the material, whereas the deviatoric stress reflects the shear effect and can be represented by the second invariant of deviatoric stress:

4 $${\mathrm{I}}_{1} = \frac{{\sigma_{1} + \sigma_{2} + \sigma_{3} }}{3}$$

5 $${\mathrm{J}}_{{2}} = \frac{{(\sigma_{1} - \sigma_{2} )^{2} + (\sigma_{2} - \sigma_{3} )^{2} + (\sigma_{3} - \sigma_{1} )^{2} }}{6}$$

where *I*_*1*_ denotes the spherical stress and,*J*_*2*_ represents the second invariant of deviatoric stress. For most coal-rock materials, deviatoric stress governs shear failure, and the peak value of the corresponding distortional energy generally coincides with the boundary of the plastic zone. Therefore, deviatoric stress is an important indicator for identifying the initiation of damage.

When the coal pillar enters the elastoplastic stage, the stress–strain relationship no longer strictly follows fully recoverable elastic behavior. Irreversible processes such as fracture propagation, sliding, and microstructural damage lead to energy dissipation, and the elastic strain energy can be expressed as:

6 $$U_{e} = \frac{{I_{1}^{2} }}{18K} + \frac{{J_{2} }}{2G}$$

where *K* denotes the bulk modulus and *G* denotes the shear modulus. To characterize the proportional relationship between elastic strain energy and energy dissipation, the dissipated energy ratio is introduced as:

7 $$\eta = \frac{{W_{{\mathrm{d}}} }}{{U_{{\mathrm{e}}} + W_{{\mathrm{d}}} }}$$

An increase in *η* from low to high reflects the mechanical evolution of different regions within the coal pillar during loading. Based on previous studies, the energy dissipation zone is generally larger than the plastic zone and may cover regions where crack initiation and propagation occur but significant plastic deformation has not yet developed. Therefore, compared with conventional plastic zone-based indicators, *η* can more comprehensively characterize the damage state of the coal pillar^20,21^.

By integrating the spherical and deviatoric stress components with the corresponding energy decomposition, the interior of the coal pillar can be divided into an elastic core zone and a plastic shell zone. In the elastic core zone, volumetric strain energy is moderate, distortional energy is relatively low, and *η* remains small, indicating an effective load-bearing structure. In contrast, in the plastic shell zone, deviatoric stress and distortional energy accumulate rapidly, *η* is relatively high, and damage evolution is dominated by energy dissipation. Variations in coal pillar width essentially alter the relative volumes and energy distributions of these two zones, thereby controlling the overall stability of the pillar. The above stress-driven energy decomposition provides a theoretical basis for numerical implementation.

### Secondary development based on the energy-transmission model

Based on the explicit finite difference method, FLAC3D updates stress–strain increments through time stepping, and the built-in plastic work can be equivalently used to represent irreversible energy dissipation of materials. This approach has been widely applied in studies on deep surrounding rock failure^22^.

**Fig. 2:**
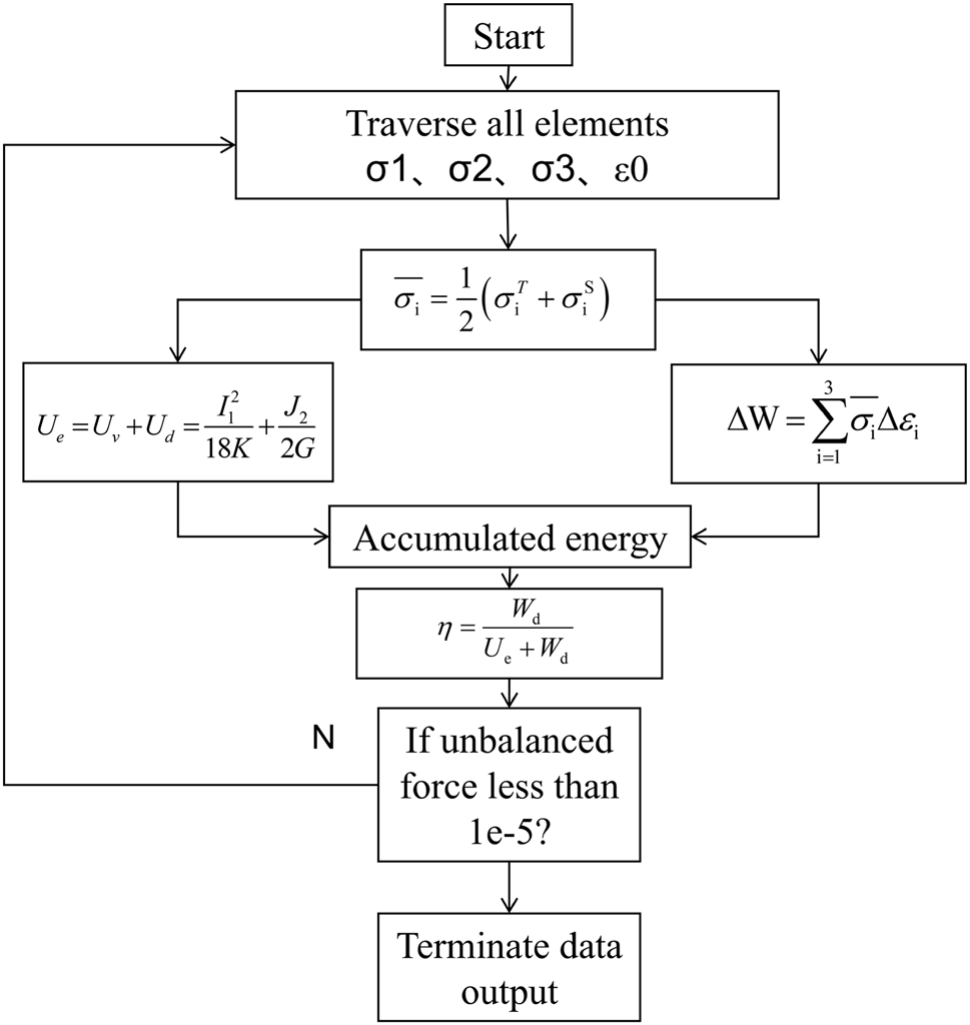
Calculation flowchart.

In this study, elastic strain energy and dissipated energy are adopted as the primary indicators for analyzing energy distribution and damage evolution. As gob-side entry driving represents a single disturbance process, the overall energy evolution can be abstracted as a sequential process of elastic elastic strain energy, dissipated energy, and subsequent static equilibrium. Accordingly, three indicators—elastic energy, plastic work, and dissipated energy ratio—are established to achieve a quantitative representation of energy evolution in numerical simulations. The specific calculation procedure is illustrated in Fig. 2.

Within a time step *t* → *t* + *Δt* , FLAC3D performs principal value decomposition of the element stress tensor:

8 $$\sigma_{1} < \sigma_{2} < \sigma_{3}$$

The corresponding strain increment can be expressed as the superposition of elastic and plastic components:

9 $$\Delta \varepsilon_{{\mathrm{i}}} = \Delta \varepsilon_{{\mathrm{i}}}^{e} + \Delta \varepsilon_{i}^{p}$$

where *Δε*_*i*_ denotes the total strain increment, ${\Delta \varepsilon }_{i}^{p}$ is the elastic strain increment, and ${\Delta \varepsilon }_{i}^{p}$ is the plastic strain increment. To reduce the influence of instantaneous fluctuations, the average principal stresses within a time step are adopted for energy calculation:

10 $$\overline{{\sigma_{{\mathrm{i}}} }} = \frac{1}{2}\left( {\sigma_{{\mathrm{i}}}^{T} + \sigma_{{\mathrm{i}}}^{{{\mathrm{T}} + 1}} } \right)$$

The energy variation of an element during a time step is obtained from the stress–strain integration:

11 $$\Delta {\mathrm{W}} = \sum\limits_{{{\mathrm{i}} = 1}}^{3} {\overline{{\sigma_{{\mathrm{i}}} }} \Delta \varepsilon_{{\mathrm{i}}} }$$

When *Δε*_*i*_ is purely elastic, the corresponding energy is stored within the element. When the plastic strain increment is nonzero, the material enters the yield regime, accompanied by irreversible processes such as microcrack propagation, particle sliding, and shear failure, and the corresponding energy is transformed into irreversible dissipated energy. As gob-side entry driving represents a typical “single disturbance-tending to equilibrium” condition, the coal pillar does not experience repeated loading–unloading cycles. Therefore, a full cyclic energy model is not required, and the recoverable post-peak energy has a negligible influence on the final stability and can be ignored. Under this condition, the accumulation of plastic strain increments can be directly used to represent the damage energy of the element.

12 $${\mathrm{W}}_{{\mathrm{d}}} = \sum\limits_{{{\mathrm{n}} = 1}}^{{N_{eq} }} {\sum\limits_{i = 1}^{3} {\overline{{\sigma_{i}^{\left( n \right)} }} \Delta \varepsilon_{i}^{p} \left( n \right)} }$$

where *N*_*eq*_ denotes the number of time steps required to reach a static equilibrium state. In this study, an unbalanced force less than 1 × 10^–5^ is adopted as the criterion for static equilibrium. Under static equilibrium conditions, the total energy of an element consists of elastic strain energy *U*_*e*_ and dissipated energy *W*_*d*_*.*

In summary, based on the explicit finite difference framework of FLAC3D, this study achieves a numerical implementation of energy transfer theory through the stress–strain increment updating mechanism, accumulation of plastic work, and the dissipated energy ratio. This approach not only enables a quantitative comparison of energy input, elastic strain energy, and energy dissipation pathways under different coal pillar widths, but also allows identification of the internal energy structure evolution of coal pillars from an elastic core to a plastic shell.

## Numerical model and parameters for gob-side entry driving

### Engineering overview

**Fig. 3:**
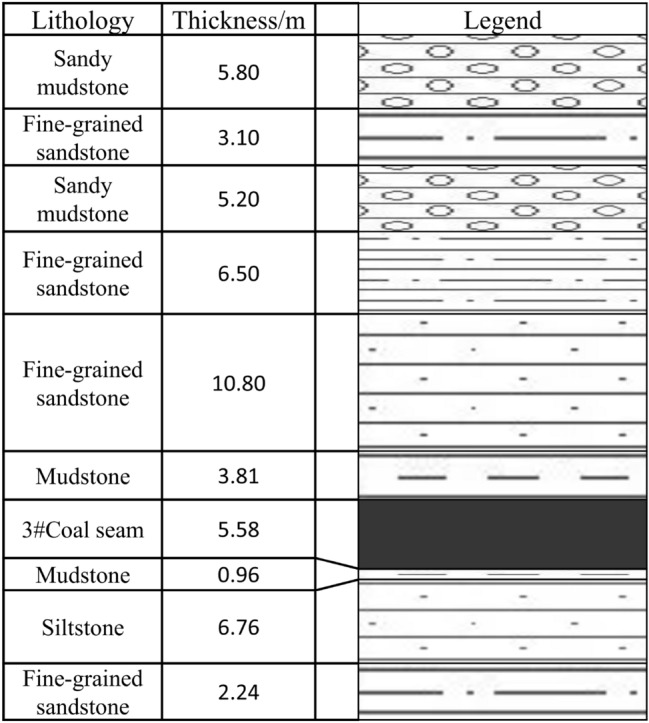
Distribution map of coal seam and rock strata.

The 6312 longwall panel of the Changping Coal Mine has a strike length of 482 m and a dip length of 260 m, with a total seam thickness of 5.6 m. The 63,123 headgate is a gob-side entry driven along the roof and adjacent to the goaf of the 6301 panel. The average burial depth is 501 m. The roadway has a rectangular cross-section measuring 5.2 m in height and 3.6 m in width. The coal seam dips at an average angle of 7°. The stratigraphic and lithological conditions are shown in Fig. 3.

### Numerical simulation model

**Fig. 4:**
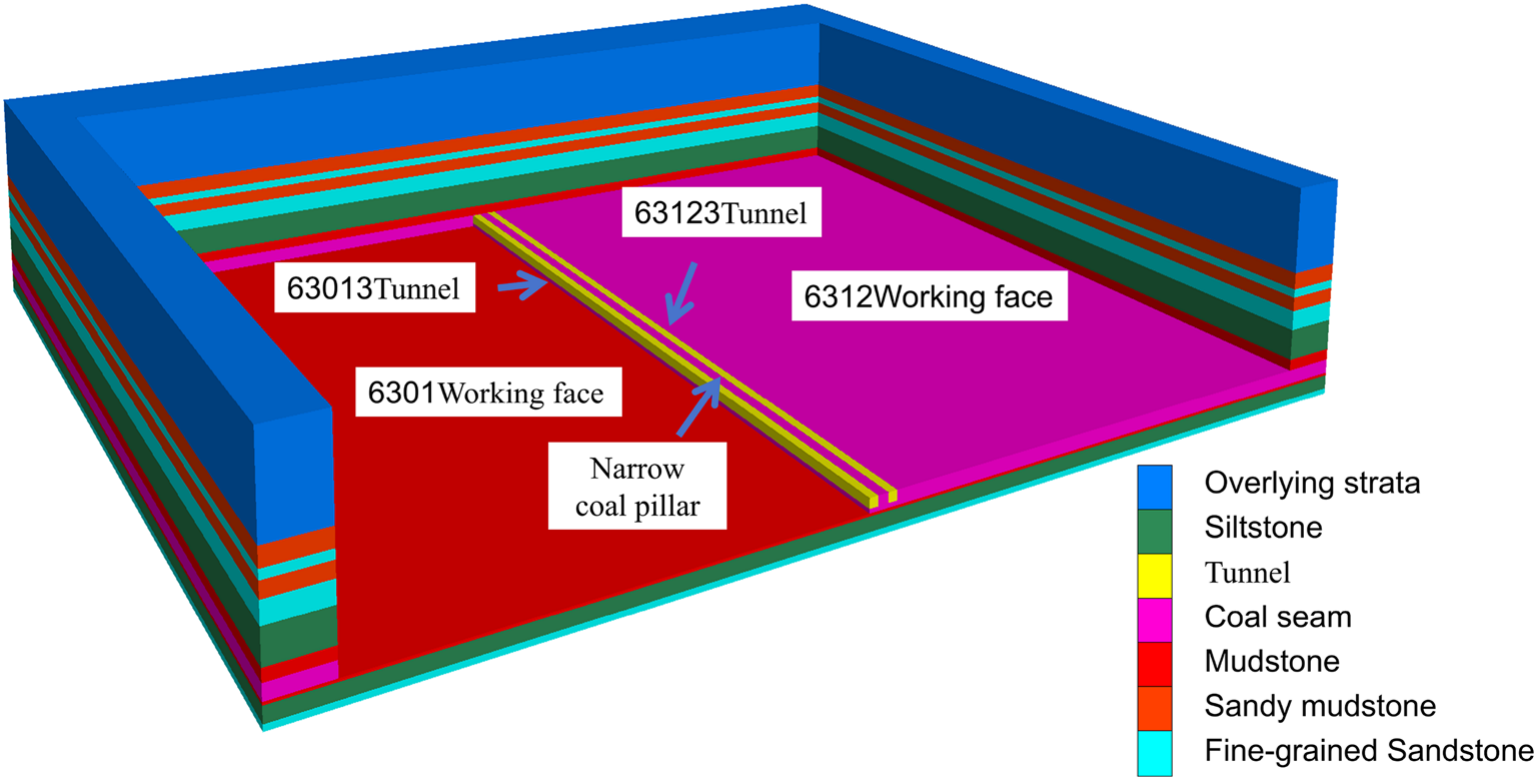
Schematic diagram of numerical simulation for 6312 workface.

To determine the appropriate narrow coal-pillar width for gob-side entry driving in the thick and soft coal seam, and to develop a corresponding support scheme, a three-dimensional FLAC3D numerical model consistent with the geological conditions of the 6312 panel was established. The overall modeling layout for the 6312 working face is illustrated in Fig. 4.

The numerical model has overall dimensions of 480 m in length, 600 m in width, and 80 m in height. The 6301 goaf region is represented with a length of 480 m and a width of 260 m. The overlying strata not explicitly modeled were replaced by an equivalent vertical load of 11 MPa applied to the top boundary, with a lateral pressure coefficient of 0.9. Fixed-displacement boundaries were assigned along the model’s sides and base. The Mohr–Coulomb constitutive model was adopted, and the mechanical parameters of the coal and rock layers are listed in Table 1. Considering the effects of weathering and hydration on the mudstone under field conditions, a strain-softening constitutive model was adopted for the mudstone. The residual cohesion was taken as 30% of the peak value, and the residual internal friction angle was set to 13°.

**Table 1:** Mechanical parameters of rock mass in numerical model.

Rock stratum	Density/kg·m^-3^	Shear modulus/GPa	Bulk modulus/GPa	Cohesion/MPa	Friction angle/(°)	Tensile strength/MPa
Overlying strata	2662	2.09	2.78	2.55	30	4.56
Siltstone	2650	3.02	5.04	9.42	42.5	5.20
Fine-grained Sandstone	2600	2.33	3.89	8.26	43.8	5.40
Mudstone	2520	0.70	1.71	2.04	37.6	0.56
Sandy mudstone	2630	1.26	2.59	5.17	37.9	1.21
Coal seam	1350	0.41	1.08	1.24	31.2	0.28

The mechanical parameters of the coal seam and surrounding rock were determined based on laboratory testing and calibrated with field data and published literature.Specifically, parameters such as elastic modulus, Poisson’s ratio, cohesion, internal friction angle, and tensile strength were obtained from experimental results and adjusted to reflect in-situ conditions.The vertical stress was applied on the upper boundary to simulate the overburden load, and the horizontal stress was determined according to the lateral pressure coefficient.The initial stress field was established according to the burial depth and geological conditions of the study area.

Because the 6301 goaf had been stabilized for more than one year by the time the 63,123 gob-side entry was driven, the analysis focuses on post-stabilization conditions. Six coal-pillar widths-4 m, 6 m, 8 m, 10 m, 12 m, and 14 m-were evaluated to examine the failure characteristics and surrounding-rock stability of the roadway.

## Comparison of mechanical responses and energy-based indicators under different coal pillar widths

### Conventional mechanical responses

**Fig. 5:**
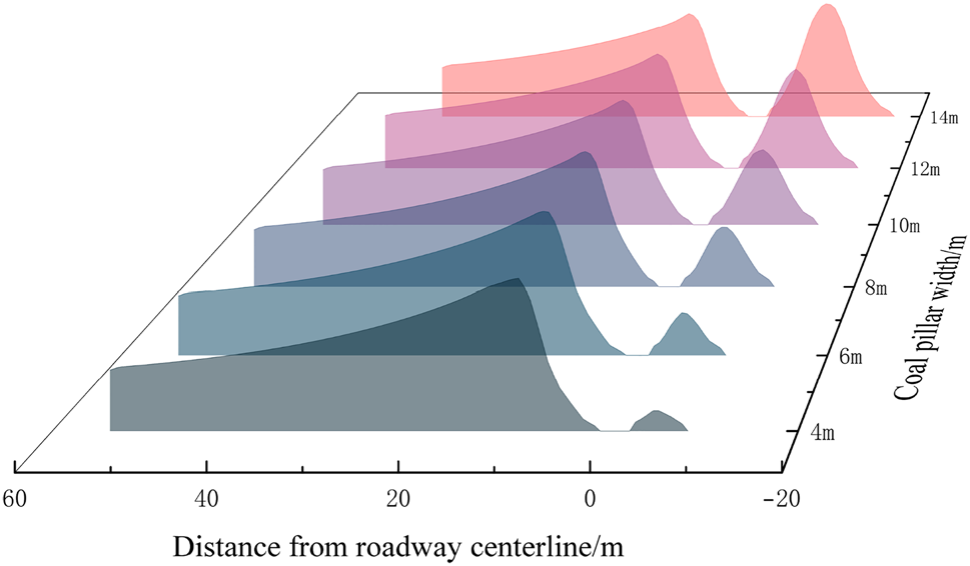
Stress variation curves.

**Fig. 6:**
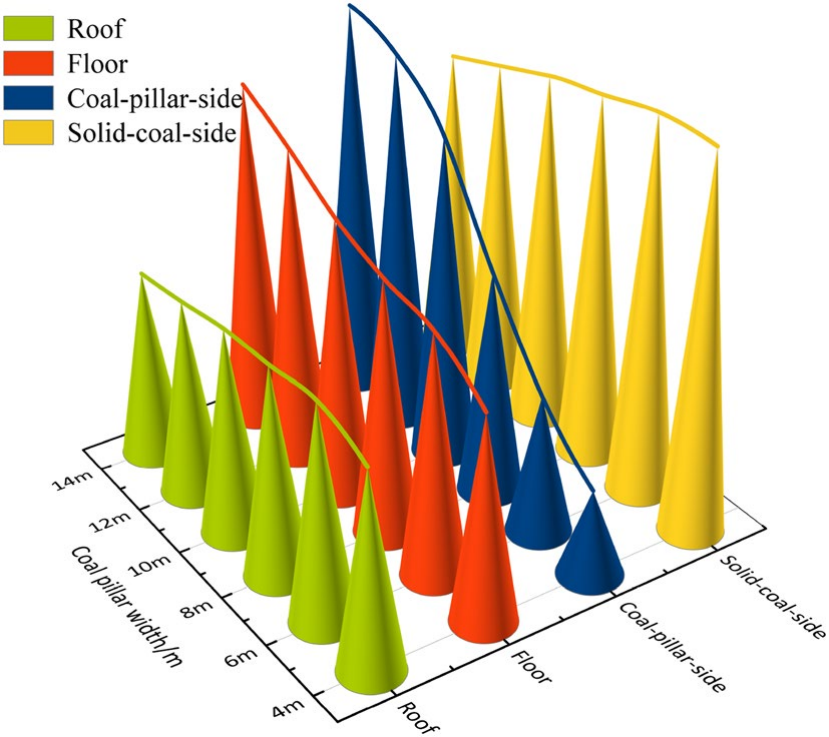
Distribution of vertical displacement.

To provide a reference for subsequent energy-based indicators, the conventional mechanical responses under different coal-pillar widths were first analyzed. The stress-width relationships are shown in Fig. 5, and the displacement distributions for each width are presented in Fig. 6.

The vertical stress within the coal pillar exhibits a single-peak distribution for all widths, but both the magnitude and location of the peak vary significantly. For widths of 4 ~ 6 m, the peak stress is relatively low and the pillar remains largely unloaded. When the width increases to 8 ~ 14 m, the peak stress rises sharply and the concentration zone shifts toward the pillar center, indicating greater load-bearing capacity but also a higher tendency for energy concentration.

Roof subsidence and rib-side convergence both show a trend of first increasing and then decreasing with pillar width: deformation is pronounced at widths of 4 m and 6 m, whereas it gradually decreases once the width exceeds 10 m. This indicates that pillar widening enhances surrounding-rock stiffness within a certain range, but excessive width leads to stress and energy concentration inside the pillar, increasing the potential for localized instability.

As the coal pillar width increases, the plastic zone within the coal pillar gradually decreases, an regions begin to appear. When the coal pillar width is relatively small, failure is dominated by shear deformation. With increasing pillar width, the failure mode evolves into a combination of shear and tensile failure. This indicates that the internal damage of the coal pillar is progressively alleviated as the pillar width increases.

Overall, stress and displacement responses can reflect the general load-bearing behavior of the pillar but fail to reveal the internal partitioning between elastic elastic strain energy and dissipated energy, nor can they identify latent energy-concentration zones. Therefore, elastic strain energy, dissipated energy, and the energy-dissipation ratio must be introduced to more accurately characterize pillar stability, failure mode, and optimal width.

### Analysis of dissipated energy and elastic strain energy

To simplify the analysis, three representative indicators are selected: elastic strain-energy density, dissipated-energy density, and the energy-dissipation ratio. The trends of volumetric and elastic strain energy are generally similar, while the distribution of distortional energy closely resembles that of dissipated energy; thus, they are not discussed further.

**Fig. 7:**
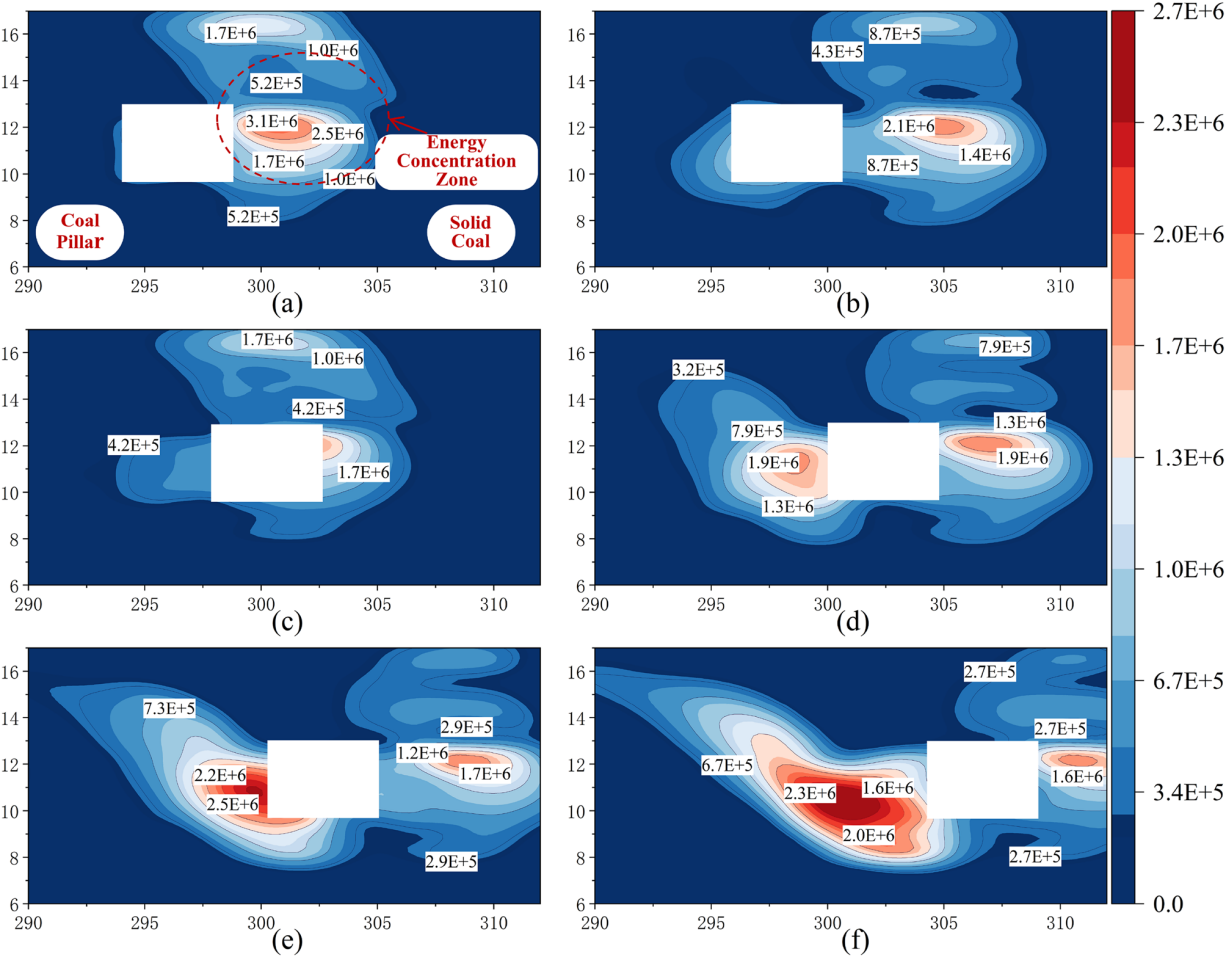
**(a)** Distribution of dissipated energy of the 4 m coal pillar; **(b)** Distribution of dissipated energy of the 6 m coal pillar; **(c)** Distribution of dissipated energy of the 8 m coal pillar; **(d)** Distribution of dissipated energy of the 10 m coal pillar;**(e)** Distribution of dissipated energy of the 12 m coal pillar; **(f)** Distribution of dissipated energy of the 14 m coal pillar.

The dissipated-energy distributions for different pillar widths are shown in Fig. 7. The results indicate that both the coal pillar and the solid-coal rib exhibit a similar pattern: a single-peak curve skewed toward the roadway side, with a dissipation concentration zone forming at the upper corner of the solid-coal rib. As pillar width increases, dissipation within the pillar gradually grows, the concentration zone in the solid-coal rib diminishes, and the high-dissipation region shifts toward the lower left area of the pillar. For widths of 4 ~ 8 m, dissipated energy inside the pillar remains low, whereas widths greater than 10 m produce a distinct dissipation concentration zone within the pillar.

This behavior is attributed to the stabilized condition of the goaf during simulation. After complete extraction of the adjacent panel, the overlying strata load is transferred to the front coal mass and the pillar, while the goaf roof collapses and compacts. During roadway excavation, the load originally borne by the coal mass shifts to the pillar and solid-coal rib, causing dissipation to concentrate along the pillar boundary. The high-dissipation zone at the upper corner of the solid-coal rib suggests that installing corner bolts would enhance support effectiveness and improve surrounding-rock stability.

**Fig. 8:**
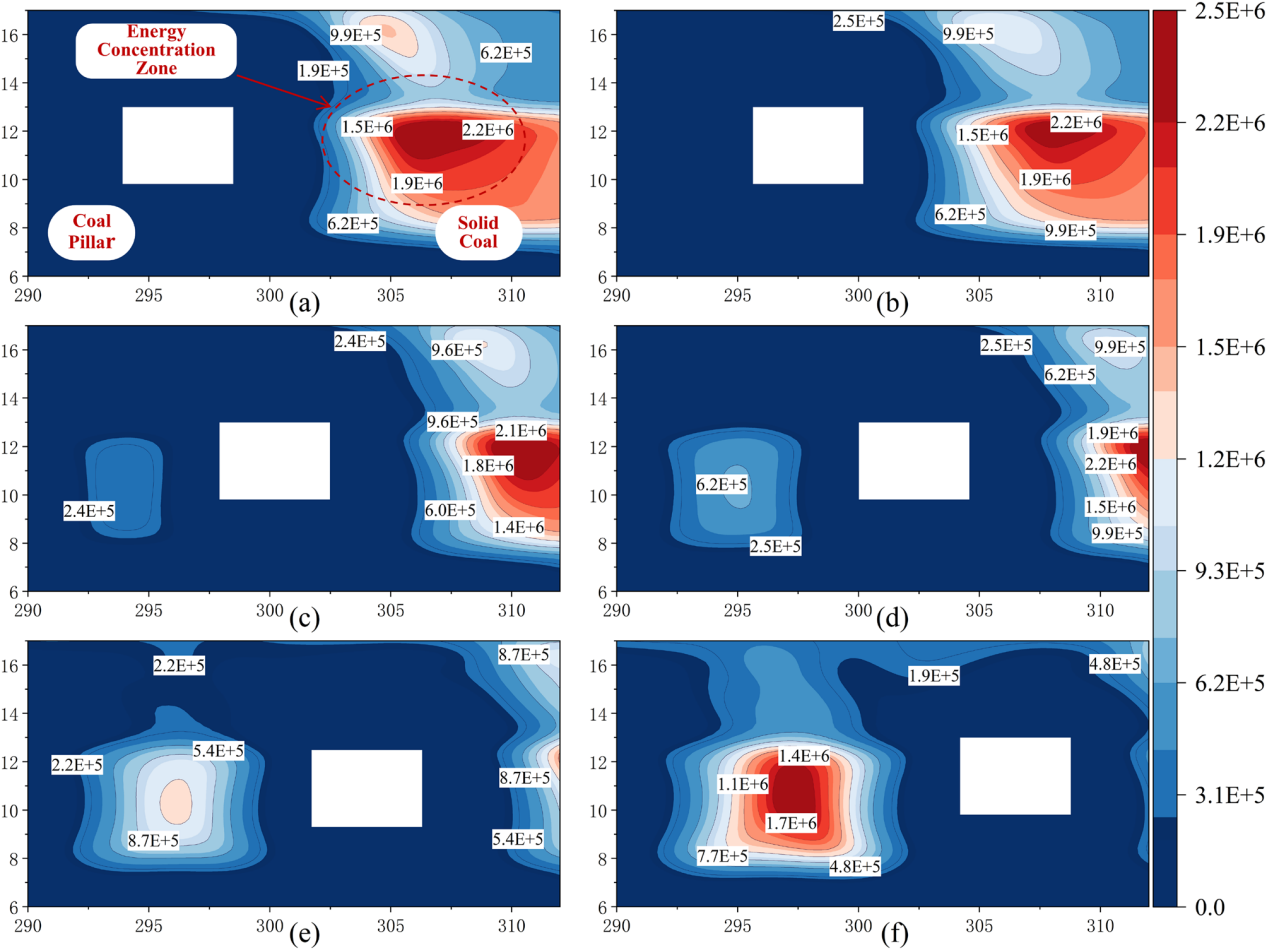
**(a)** Distribution of elastic strain energy of the 4 m coal pillar; **(b)** Distribution of elastic strain energy of the 6 m coal pillar; **(c)** Distribution of elastic strain energy of the 8 m coal pillar; **(d)** Distribution of elastic strain energy of the 10 m coal pillar; **(e)** Distribution of elastic strain energy of the 12 m coal pillar; **(f)** Distribution of elastic strain energy of the 14 m coal pillar.

The elastic strain-energy distributions for different pillar widths are shown in Fig. 8. The coal pillar and the solid-coal rib exhibit similar patterns. As pillar width increases, elastic strain energy in the solid-coal rib decreases, whereas the pillar interior is evident a progressive increase, with the concentration zone shifting toward the pillar side. For widths of 4 ~ 8 m, elastic strain energy remains low overall, while widths of 10 ~ 14 m produce a pronounced concentration zone inside the pillar. Such localized elastic-energy accumulation may induce an elevated risk of coal-wall burst, indicating the need to avoid excessive internal energy buildup. The volumetric and distortional energy densities follow trends similar to those of the elastic strain energy, increasing with pillar width before stabilizing .

**Fig. 9:**
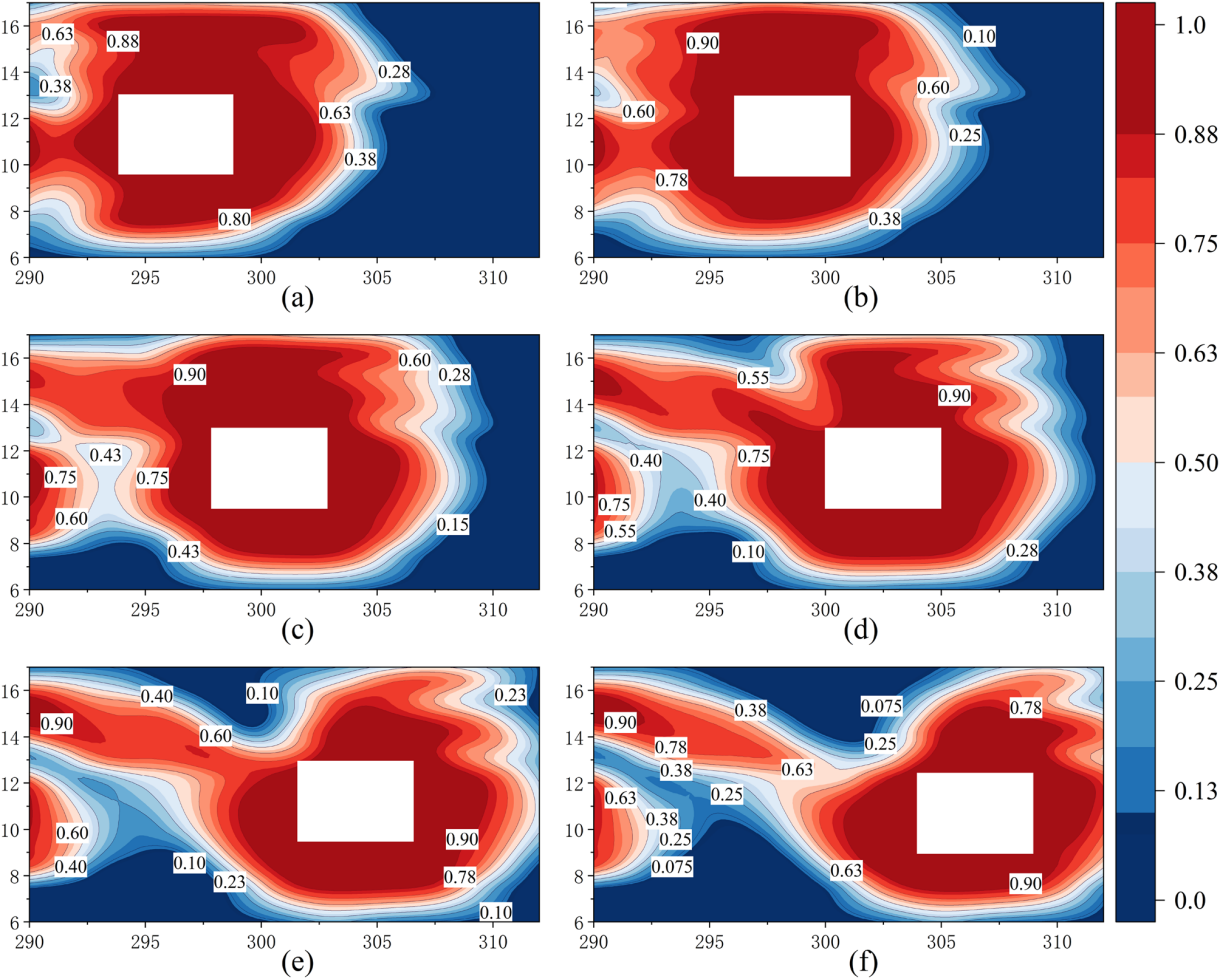
**(a)** Distribution of dissipated energy ratio of the 4 m coal pillar; **(b)** Distribution of dissipated energy ratio of the 6 m coal pillar; **(c)**Distribution of dissipated energy ratio of the 8 m coal pillar; **(d)** Distribution of dissipated energy ratio of the 10 m coal pillar; **(e)** Distribution of dissipated energy ratio of the 12 m coal pillar; **(f)** Distribution of dissipated energy ratio of the 14 m coal pillar.

The energy-dissipation ratio distributions for different pillar widths are shown in Fig. 9. The highest values occur along both sides of the pillar and gradually decrease toward the center. Elevated η values also appear around the roadway perimeter, while the solid-coal rib is observed a reduction in *η* with increasing distance from the roadway surface. At a width of 4 m, the dissipation ratio within the pillar exceeds 0.9 throughout, indicating a fully developed plastic zone and low overall strength. For widths of 6 ~ 14 m, the peak *η* progressively decreases and a relatively elastic-dominated core, implying enhanced pillar strength and a compressed plastic-dissipation zone. This shift reflects improved energy-storage capacity, although coal loss increases with width.

**Fig. 10:**
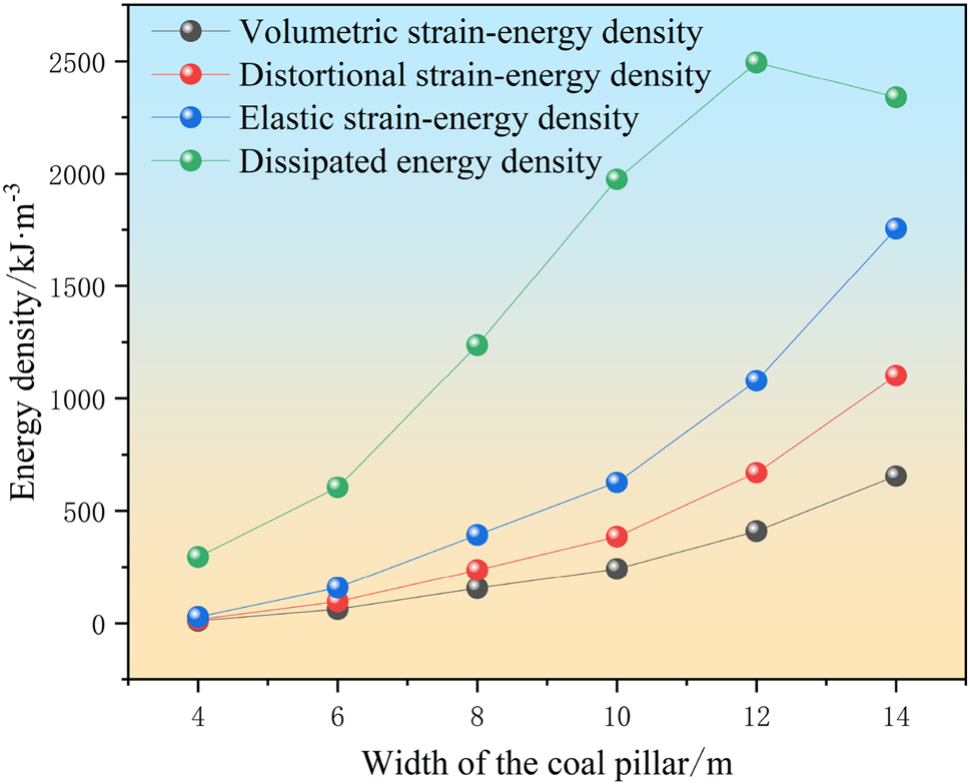
Variation of peak energy in coal pillars with pillar width.

These results demonstrate that the energy-dissipation ratio *η* effectively captures the transition of the coal pillar from elastic behavior to elastoplastic deformation and eventual failure, providing a quantitative basis for optimizing pillar width and designing roadway support systems.

The peak energy values inside the coal pillar for different widths are shown in Fig. 10. The volumetric strain-energy density, distortional-energy density, and elastic strain-energy density all increase with pillar width, and their growth accelerates significantly once the width exceeds 10 m. At a width of 4 m, the peaks of these three energy types are 10.8 kJ/m^3^, 16.9 kJ/m^3^, and 27.7 kJ/m^3^, respectively. When the width increases to 14 m, the corresponding values rise to 653.7 kJ/m^3^, 1101.4 kJ/m^3^, and 1755.1 kJ/m^3^ approximately 59, 64, and sixfold increases.

Dissipated-energy density increases with pillar width from 4 to 12 m but begins to decrease when the width exceeds 12 m. At a width of 4 m, the dissipation peak is 294.6 kJ/m^3^; it rises to 2494.9 kJ/m^3^ at 12 m, an increase of about 7.5 times. Meanwhile, both the dissipation ratio and the distortional-energy ratio in the pillar center decrease with increasing width. The dissipation ratio drops from 0.91 to 0.16, and the distortional-energy ratio decreases from 0.63 to 0.60, indicating a gradual transition of the pillar from a plastic-dominated state to an elastic-dominated state.

### Comparison between energy-based analysis and conventional analysis

**Fig. 11:**
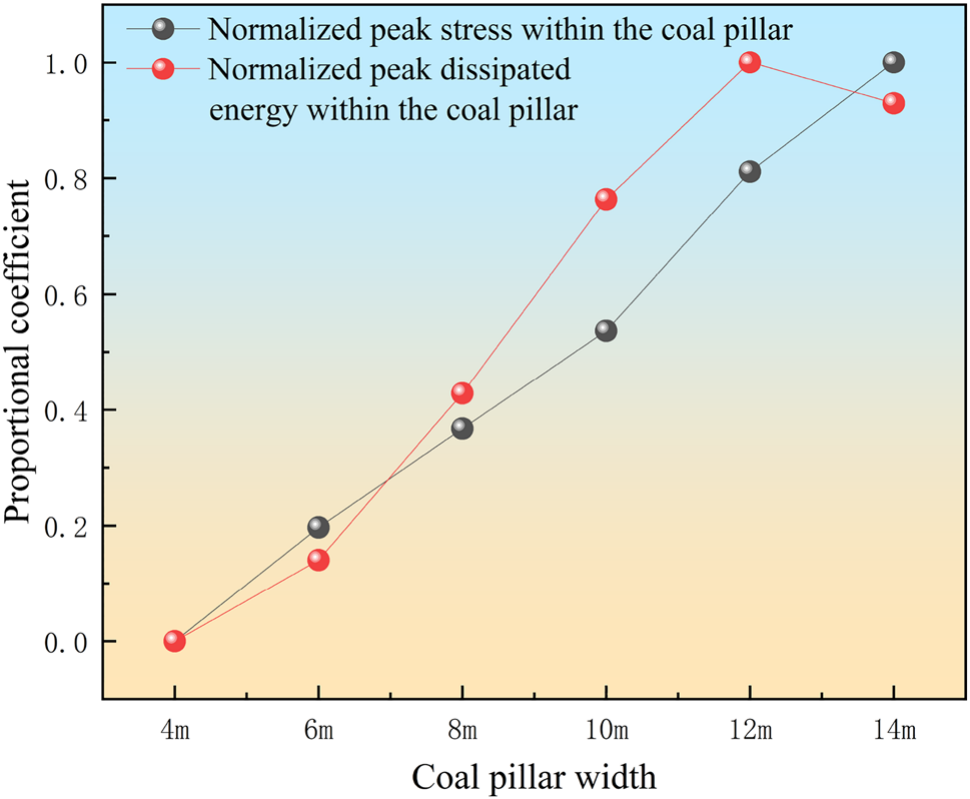
Comparison diagram of peak stress and peak dissipated energy.

The peak stress and peak dissipated energy within the coal pillar were extracted and normalized. The relationships between the normalized peak stress, peak dissipated energy, and coal pillar width are shown in Fig. 11.

The results indicate that the peak stress within the coal pillar increases linearly with increasing pillar width, without a distinct inflection point. In contrast, the peak dissipated energy within the coal pillar exhibits an overall S-shaped increasing trend as the pillar width increases. When the pillar width ranges from 4 to 6 m, the growth rate of the normalized peak dissipated energy is relatively slow, whereas it increases continuously in the range of 6 ~ 12 m. This indicates that selecting a coal pillar width of 6 m can effectively avoid the rapid-increase stage of dissipated energy.

**Fig. 12:**
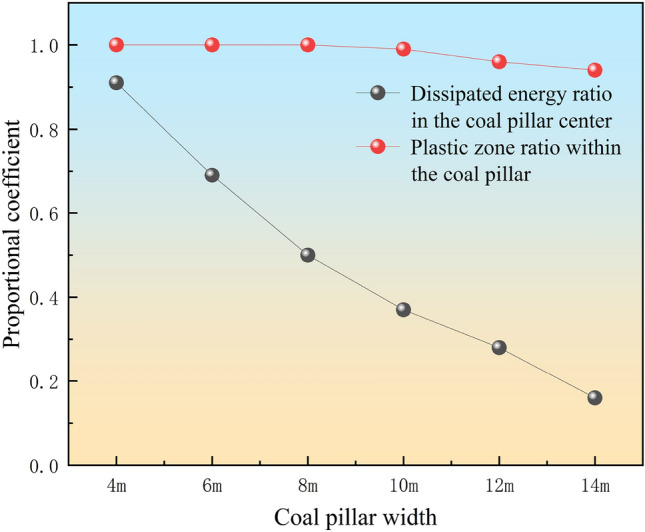
Comparison diagram of plastic zone proportions and dissipated energy ratios.

A comparison between the proportion of the plastic zone within the coal pillar and the dissipated energy ratio is presented in Fig. 12. The plastic zone proportion is defined as the percentage of the damaged area within the considered pillar width relative to the total coal pillar area. The results indicate that the plastic zone proportion decreases with increasing pillar width, with the normalized value gradually decreasing from 1 to 0.94. However, the overall rate of change is relatively small, making it difficult to identify an optimal pillar width based solely on the plastic zone proportion, especially at smaller widths. In contrast, the dissipated energy ratio within the coal pillar decreases markedly with increasing pillar width. When the pillar width ranges from 4 to 12 m, the reduction rate of the dissipated energy ratio progressively diminishes, and the dissipated energy ratio drops below 0.7 at a pillar width of 6 m.Similar conclusions have been reported in previous energy-based numerical studies, where energy evolution parameters were shown to be more sensitive than stress or plastic zone indicators in characterizing coal pillar damage processes^15^.

Integrated analysis of stress, displacement, and energy evolution indicates that the stability differences among pillar widths are primarily governed by load-bearing capacity and the degree of energy evolution characteristics. From an energy perspective, the dissipation ratio η reveals the transition from elastic strain energy to energy dissipation under external loading. As deformation develops, η typically increases from near zero in the elastic stage, rises during plastic deformation due to crack propagation and shear-slip dissipation, and then decreases as the plastic zone compacts and the system approaches a new equilibrium-indicating a shift back toward elastic dominance.

Clear distinctions emerge among pillar widths. For a width of 4 m, *η* exceeds 0.9 throughout the pillar, the plastic zone is continuous, and the structure remains in a high-dissipation, low-capacity state. At 6 m, a pronounced elastic core develops, *η* decreases to approximately 0.67, the plastic zone contracts, and both elastic and distortional energies increase, yielding a balanced coordination between storage and dissipation. When the width increases to 8 ~ 14 m, the load-bearing capacity continues to rise, but the peak energy values grow sharply, producing energy concentration and high elastic accumulation in the pillar center-conditions prone to local instability or local instability risks-while also resulting in unnecessary coal loss.

Compared with traditional evaluation methods, the energy-based approach offers several distinct advantages. Deformation and failure processes are fundamentally governed by energy accumulation and dissipation. Therefore, energy analysis provides a more intrinsic description of instability mechanisms. In addition, the evolution of elastic strain energy and dissipated energy allows for the differentiation between stable and unstable states. Compared with stress or displacement indicators, energy-based parameters exhibit higher sensitivity to instability and can capture early-stage failure characteristics. Therefore, the energy-based approach can serve as an effective complement to conventional methods in evaluating coal pillar stability.

Therefore, based on the distribution of the dissipated energy ratio and the evolution characteristics of energy peaks, the 4 m coal pillar is characterized by excessive energy dissipation and insufficient load-bearing capacity, indicating a relatively weak and unstable state. In contrast, coal pillar widths ranging from 8 to 14 m exhibit pronounced energy concentration, suggesting significant energy accumulation within the pillar, which may increase the potential risk of instability under mining disturbance. When the coal pillar width is 6 m, both stress concentration and energy evolution remain at relatively moderate levels, and a more stable energy distribution pattern is observed. Therefore, considering mechanical response, energy evolution characteristics, and economic efficiency, the 6 m coal pillar is considered to achieve the most favorable balance and can be regarded as the optimal width under the geological conditions of this site. This result further demonstrates that the proposed energy-based analysis provides a reliable basis for coal pillar width optimization.

## Field application

Based on the comprehensive numerical analysis results, after determining 6 m as the optimal coal pillar width, it is necessary to further control local energy concentration in the surrounding rock to verify the engineering feasibility of energy regulation under this width condition. Energy analysis indicates that although the 6 m coal pillar as a whole remains in an elastoplastic equilibrium state, certain degrees of energy concentration still exist in the rib sides and roof corner regions. Accordingly, a combined bolt-mesh-grouting-shotcrete support system is introduced under the optimal coal pillar width condition. By enhancing the overall confinement and bearing capacity of the surrounding rock, this support system mitigates local high-energy zones and optimizes energy distribution, thereby achieving coordinated stability between the coal pillar and the roadway surrounding rock.

### Support scheme

**Fig. 13:**
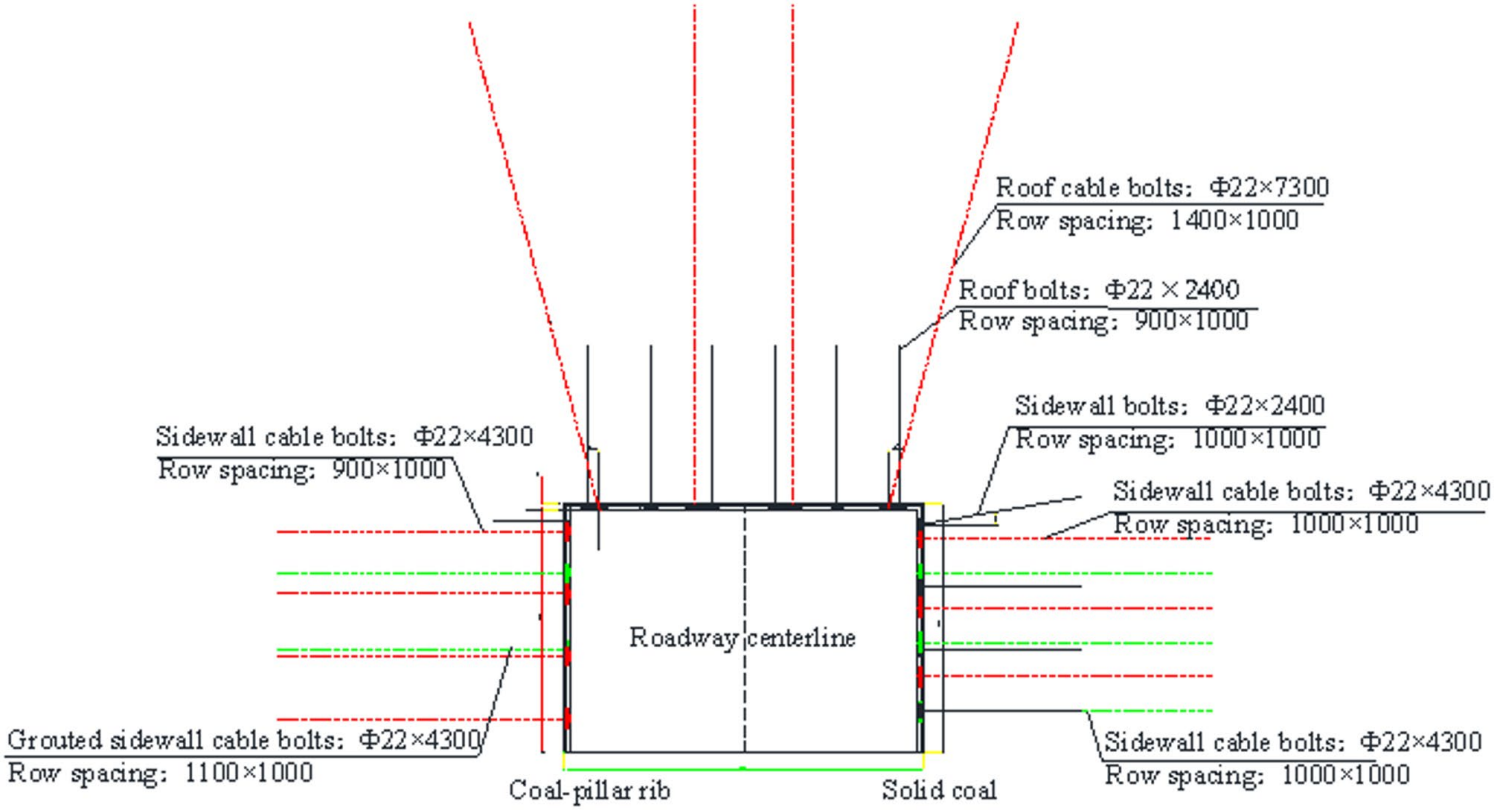
Support design of the 63,123 roadway along the 63,123.

The support scheme for the 63,123 roadway is shown in Fig. 13. Reinforcing steel mesh is installed on the roadway roof and ribs, followed by shotcrete spraying. An integrated bolt-mesh-grouting-shotcrete combined support system is adopted overall.

### Simulation results of pillar support

**Fig. 14:**
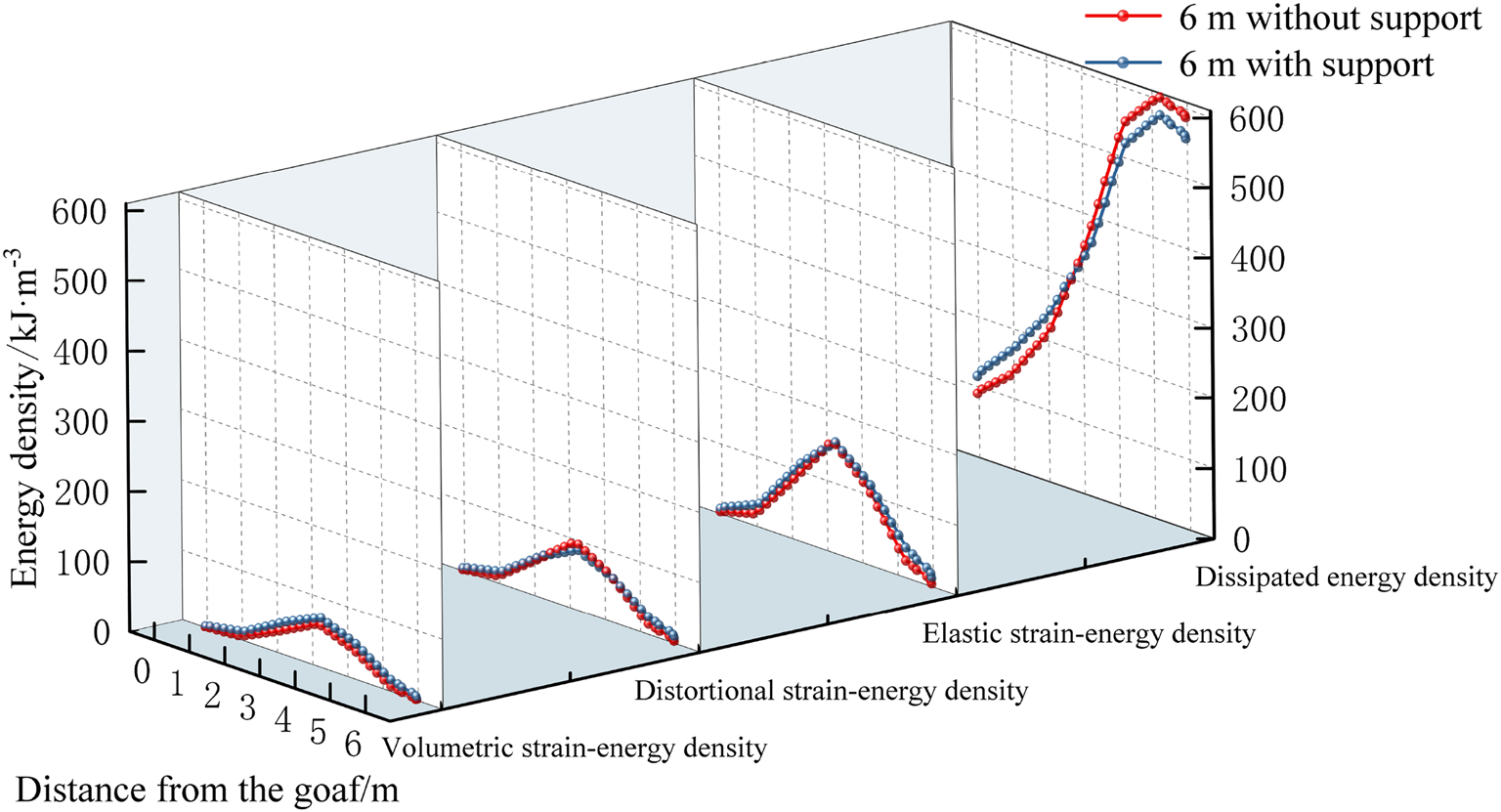
Comparison of energy characteristics for 6 m coal pillar with and without support.

The energy evolution of the 6 m coal pillar under supported and unsupported conditions is shown in Fig. 14. After support installation, both the volumetric strain energy density and the peak value of elastic strain energy *U*_*e*_ within the coal pillar increase, while the distortional energy density and the peak value of dissipated energy *W*_*d*_ decrease slightly. These results indicate that the support system effectively enhances the overall elastic strain energy capacity of the coal pillar and weakens localized dissipated energy.

**Fig. 15:**
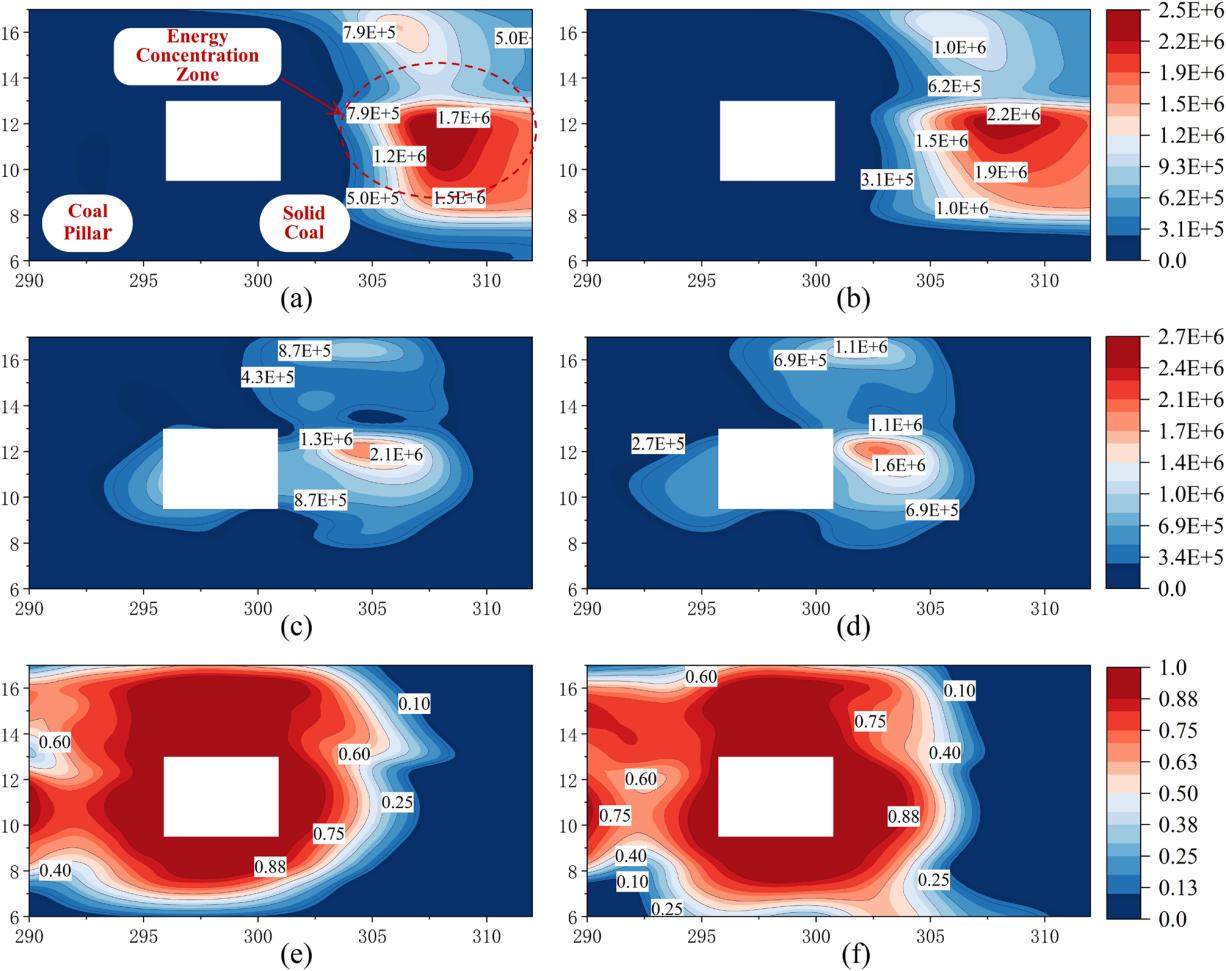
**(a)** Distribution of elastic strain energy of the 6 m coal pillar without support; **(b)** Distribution of elastic strain energy of the 6 m coal pillar with support; **(c)** Distribution of dissipated energy of the 6 m coal pillar without support; **(d)** Distribution of dissipated energy of the 6 m coal pillar with support; **(e)** Distribution of dissipated energy ratio of the 6 m coal pillar without support; **(f)** Distribution of dissipated energy ratio of the 6 m coal pillar with support.

The energy contour maps of the 6 m coal pillar under supported and unsupported conditions are shown in Fig. 15. The results indicate that after support installation, the high dissipated energy zone in the central region of the coal pillar is significantly weakened, the distribution of elastic strain energy *U*_*e*_ becomes more uniform, and the extent of the plastic zone is markedly reduced. By enhancing the overall confinement and bearing capacity of the surrounding rock, the combined support system regulates the internal energy distribution of the coal pillar, mitigates local energy concentration, and promotes a gradual transition of the coal pillar from a plastic dissipation-dominated state to a stable state dominated by elastic elastic strain energy.

In summary, the combined support system constrains plastic deformation, enhances the overall stiffness, and optimizes energy transfer, thereby promoting the transformation of coal pillar energy from localized dissipation to overall elastic strain energy. The energy contour maps further confirm this energy regulation effect: after support implementation, dissipated energy in the central region of the coal pillar is significantly reduced, and the distribution of elastic strain energy becomes more uniform. These results indicate that the proposed support measure not only provides effective load-bearing and deformation control, but also substantially improves the stability of the surrounding rock from an energy perspective.

### Field monitoring

**Fig. 16:**
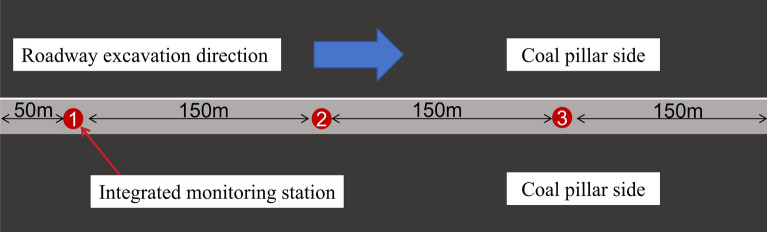
Layout of mine pressure monitoring station.

To verify the validity of the numerical simulation results, the optimized coal pillar width and support scheme were implemented in the field, and comprehensive monitoring stations were arranged at different locations along the roadway excavation direction. Three comprehensive monitoring stations were arranged along the roadway. Monitoring Station No. 1 was located 50 m from the starting point of the roadway test, Station No. 2 was located 200 m from the starting point, and Station No. 3 was located 350 m from the starting point. The plan view of the monitoring station layout is shown in Fig. 16.

**Fig. 17:**
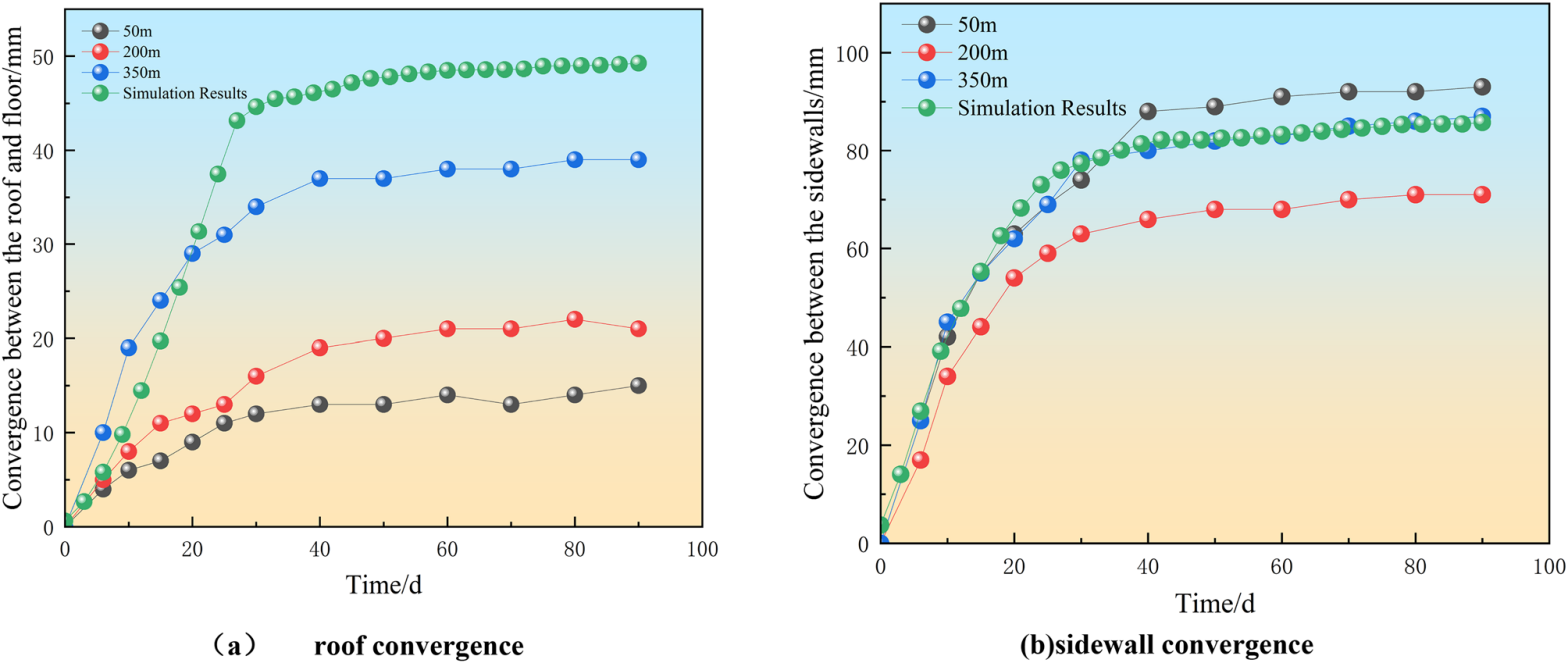
Convergence measurements of the roof and ribs at different monitoring stations during excavation.

The convergence of the roof-floor and sidewalls at different monitoring stations during excavation is presented in Fig. 17. Both the field monitoring results and the numerical simulation show a similar deformation evolution pattern, characterized by a rapid increase in the early stage followed by gradual stabilization. For sidewall convergence, the simulated final value is close to that measured at the 350 m monitoring station, while some differences remain compared with the 50 m and 200 m stations. For roof-floor convergence, the numerical result is generally higher than the monitored values, although the overall development trend is similar. These discrepancies may be related to the heterogeneity of the surrounding rock mass, differences in in-situ support conditions, and the simplification adopted in the numerical model. Nevertheless, both field and simulation results indicate that the deformation tends to stabilize after excavation, and no obvious floor heave, roof separation, or rib instability was observed, suggesting that the proposed coal pillar width and support scheme are generally reasonable for engineering application.

## Conclusion

This study analyzes the response of surrounding rock under gob-side entry driving disturbances from an energy perspective. Based on FLAC3D numerical simulations, the energy distribution, damage evolution, and their effects on surrounding rock stability under different coal pillar widths are investigated. The main conclusions are as follows:

1. Gob-side entry driving disturbances lead to rapid release of elastic strain energy in the surrounding rock on the gob side, causing the coal pillar to gradually evolve into the primary unit for energy bearing and redistribution within the system. Elastic strain elastic strain energy and dissipated energy coexist within the coal pillar, and their relative proportions vary significantly with structural scale, reflecting the evolution of the coal pillar bearing mechanism and stability state.
2. Coal pillar width, as a key controlling parameter, significantly influences the spatial distribution pattern of internal energy. As the coal pillar width increases from a small value to a moderate range, a core bearing zone dominated by elastic elastic strain energy and constrained plastic dissipation gradually forms in the central region of the coal pillar. The dissipated energy ratio decreases markedly, and the system transitions from a dissipation-dominated state to an elastoplastic coordinated state. With further increases in pillar width, elastic strain energy continues to accumulate in the central region, forming an energy concentration zone. As a result, the degree of internal energy distribution imbalance increases, accompanied by a higher potential instability risk.
3. A comparative analysis indicates that conventional indicators based on stress, displacement, and plastic zone distribution primarily reflect the bearing capacity and failure extent of the coal pillar, while exhibiting limited capability in identifying stability transition processes. In contrast, the dissipated energy ratio enables a direct characterization of the transition from dissipated energy–dominated behavior to elastic strain energy–dominated behavior. Its variation with coal pillar width exhibits clear stage-wise characteristics, suggesting that it can serve as an effective complementary indicator for evaluating coal pillar stability in gob-side entry driving.
4. Under the geological and engineering conditions considered in this study, the energy analysis results indicate that coal pillars with moderate widths exhibit more coordinated energy distribution characteristics. Field monitoring results show deformation evolution trends consistent with those obtained from numerical simulations, providing indirect support for the rationality of the proposed coal pillar design. These findings suggest that energy-based indicators can serve as an effective supplementary approach for evaluating coal pillar stability.

In summary, this study establishes an energy-distribution-based analytical framework for evaluating coal pillar stability in gob-side entry driving and introduces the dissipated energy ratio as an energy indicator for characterizing transitions in the internal mechanical state of coal pillars. From an energy perspective, this approach complements traditional mechanical indicators in stability assessment and provides a reference for coal pillar width design and surrounding rock control under similar geological conditions.

## Data Availability

The partial or all data generated or used during the research process can be provided by the corresponding author upon request.
